# CVD risk in non-albuminuric chronic kidney disease in hypertensive, non-diabetic subjects: A *post-hoc* analysis from SPRINT

**DOI:** 10.3389/fcvm.2022.977938

**Published:** 2022-12-07

**Authors:** Chang-Sheng Sheng, Dan Wang, Jiangzi Yuan, Yi Cheng, Siming Sun, Yulin Yang, Ya Miao, Weiming Wang, Jingyan Tian, Zachary T. Bloomgarden

**Affiliations:** ^1^Department of Cardiovascular Medicine, State Key Laboratory of Medical Genomics, Shanghai Key Laboratory of Hypertension, Shanghai Institute of Hypertension, Ruijin Hospital, Shanghai Jiao Tong University School of Medicine, Shanghai, China; ^2^Renal Division, Renji Hospital, Shanghai Jiao Tong University School of Medicine, Shanghai, China; ^3^Department of Endocrine and Metabolic Diseases, State Key Laboratory of Medical Genomics, Ruijin Hospital, Clinical Trial Center, Shanghai Institute of Endocrine and Metabolic Diseases, Shanghai Jiao Tong University School of Medicine, Shanghai, China; ^4^Renal Division, Ruijin Hospital, Shanghai Jiao Tong University School of Medicine, Shanghai, China; ^5^Division of Endocrinology, Diabetes, and Bone Disease, Department of Medicine, Icahn School of Medicine at Mount Sinai, New York, NY, United States

**Keywords:** albuminuria, hypertension, SPRINT trial, chronic kidney disease, cardiovascular risk factors

## Abstract

**Introduction:**

The risks associated with non-albuminuric chronic kidney disease (CKD) have been investigated in diabetes mellitus but not in hypertensive patients. The objective of this study was to investigate the risks associated with non-albuminuric CKD in treated hypertensive patients in the Systolic Blood Pressure Intervention Trial (SPRINT) population.

**Methods:**

Based on baseline albuminuria status (urine albumin/creatinine ratio [UACR], ≥30 or <30 mg/g) and the levels of estimated glomerular filtration rate ([eGFR], ≥60, 45–59, or <45 mL/min/1.73 m^2^), participants were classified into six subgroups to assess the risks associated with the primary outcome and mortality. The primary composite outcome was myocardial infarction, other acute coronary syndromes, stroke, heart failure, or mortality from cardiovascular causes.

**Results:**

During a median follow-up of 3.26 years in 8,866 hypertensive patients, there were 352 deaths and 547 participants with the primary outcome. In adjusted Cox regression analysis using non-CKD and non-albuminuria (eGFR ≥60 mL/min/1.73 m^2^ combined with UACR <30 mg/g) as reference, albuminuria whether combined with CKD or not, showed significantly higher risk of both primary outcome and all-cause mortality in the total population. Whereas, non-albuminuria only combined with eGFR <45 mL/min/1.73 m^2^ showed significantly higher risk of both primary outcome and all-cause mortality in the intensive-therapy group.

**Discussion:**

Non-albuminuric CKD did have higher risk of all-cause and CVD mortality only if the eGFR <45 mL/min/1.73 m^2^. Increased albuminuria conferred higher risk of primary outcome and all-cause mortality irrespective the levels of eGFR.

**Clinical trial registration:**

ClinicalTrials.gov, number: NCT01206062.

## Introduction

Diabetes mellitus and hypertension are the two leading risk factors of chronic kidney disease (CKD), in which increased albuminuria and decreased estimated glomerular filtration rate (eGFR) are the major determinants (1, 2). Previous studies on diabetic kidney disease showed a decreased prevalence of albuminuria and increased prevalence of decreased eGFR compared to those without diabetes (3, 4). At present, non-albuminuric renal impairment (i.e., non-albuminuric diabetic kidney disease [DKD]) has become prevalent in diabetes mellitus, and might play an important role of CKD progression and carry higher risk of outcomes (5–9). Indeed, non-albuminuric CKD did have higher risks of death or major cardiovascular events, but was not comparable to those with albuminuria (albuminuric CKD or albuminuric non-CKD) in the Action to Control Cardiovascular Risk in Diabetes (ACCORD) trial (10). However, a recent observational study in Japan failed to find non-albuminuric CKD had higher risks of mortality, cardiovascular disease (CVD) events, or renal function decline than those in no-CKD (11). It seems that albuminuria, rather than reduced eGFR, is the main driver for CKD progression and outcomes in diabetes mellitus. In fact, some studies have indicated that any degree of albuminuria is a risk factor for cardiovascular events in individuals with or without diabetes mellitus (12).

After diabetes mellitus, hypertension is the second important cause of CKD, and a major risk factor for the development and progression of CKD (1, 2, 13, 14). In patients with CKD, masked hypertension and elevated nighttime BP are common, and are associated with a lower eGFR and higher levels of albuminuria (15). A recent large meta-analysis has shown a significant reduction in all-cause mortality following BP reduction in patients with CKD (16). Reduction of albuminuria has also been considered as a therapeutic target in hypertensive patients (17). However, the risks associated with non-albuminuric CKD in non-diabetic hypertensive patients have rarely been investigated.

The Systolic Blood Pressure Intervention Trial (SPRINT) was the largest randomized clinical trial to date, to test the efficacy of a target systolic blood pressure goal of <120 mmHg compared to a standard blood pressure goal of <140 mmHg on lowering the risk of clinical outcomes in hypertensive patients free of diabetes mellitus (18), which enabled us to investigate the risks associated with various eGFR subgroups and albuminuria categories, as well as the non-albuminuric CKD. In the present study, we employed the data from the SPRINT trial to investigate the risks associated with non-albuminuric CKD in hypertensive patients.

## Research design and methods

### Study population

**Figure 1:**
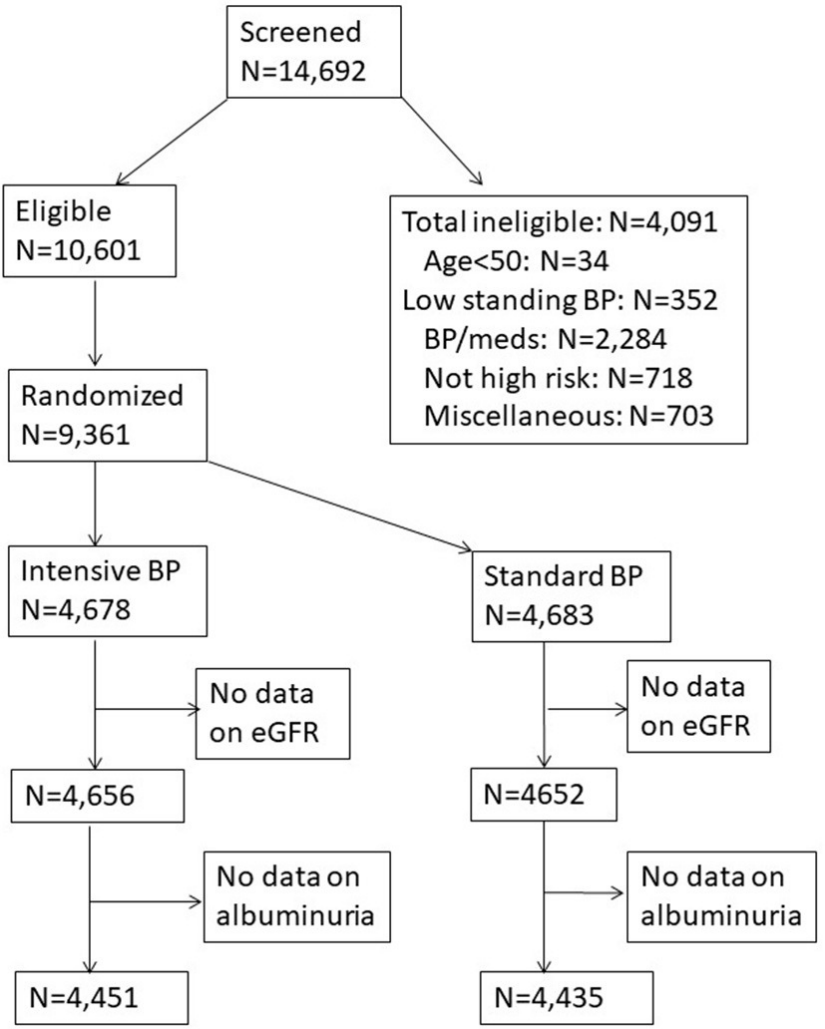
Flow chart presenting selection of the SPRINT participants for the current analysis.

SPRINT was a multicenter randomized clinical trial sponsored by the National Heart, Lung, and Blood Institute (NHLBI), the National Institute for Diabetes and Digestive and Kidney Diseases, National Institute on Aging (NIA), and the National Institute of Neurological Disorders and Stroke (NINDS) (online study protocols: https://biolincc.nhlbi.nih.gov/studies/sprint/). A thorough description of the rationale and design of SPRINT has been published (18). SPRINT was the largest randomized clinical trial to date, to test the efficacy of a target systolic blood pressure goal of <120 mmHg compared to a standard blood pressure goal of <140 mmHg on lowering the risk of clinical outcomes. SPRINT was approved by Institutional Review Boards at participating sites and all participants provided written informed consent. Figure 1 presents the flow chart of the study population selected for our analysis.

### Measurements

Urine albumin/creatinine ratio (UACR) was performed using a spot urine sample obtained at the baseline visit and measured in milligrams of albumin/gram of creatinine (mg/g). Estimated GFR (eGFR) was calculated in mL/min/1.73 m^2^ using the serum creatinine level obtained at the baseline visit. The 2021 Chronic Kidney Disease Epidemiology Collaboration equation (2021 CKD-EPI creatinine-based equations to estimate GFR without race) was used for eGFR calculating (18). Detailed inclusion and exclusion criteria have been described previously (19). Both UACR and serum creatinine were measured at the SPRINT central laboratories.

Albuminuria was defined as a UACR ≥30 mg/g. CKD was defined as eGFR <60 mL/min/1.73 m^2^. Based on baseline albuminuria status and the levels of eGFR (≥60, 45–59, or <45 mL/min/1.73 m^2^), participants were classified into six subgroups: non-albuminuric non-CKD (UACR <30 mg/g and eGFR ≥60 mL/min/1.73 m^2^), non-albuminuric CKD 3a (UACR <30 mg/g and eGFR of 45–59 mL/min/1.73 m^2^), non-albuminuric CKD 3b/4/5 (UACR <30 mg/g and eGFR of <45 mL/min/1.73 m^2)^, albuminuric non-CKD (UACR ≥30 mg/g and eGFR ≥60 mL/min/1.73 m^2^), albuminuric CKD 3a (UACR ≥30 mg/g and eGFR of 45–59 mL/min/1.73 m^2^), and albuminuric CKD 3b/4/5 (UACR ≥30 mg/g and eGFR of <45 mL/min/1.73 m^2^). A 10-year risk of cardiovascular disease was calculated on the basis of the Framingham risk score.

### Study outcomes

The primary composite outcome was considered to have occurred with myocardial infarction, acute coronary syndrome not resulting in myocardial infarction, stroke, acute decompensated heart failure, or death from cardiovascular causes. Total mortality and cardiovascular mortality refer to death from any cause and to death from cardiovascular cause.

Pre-specified subgroups of interest for primary outcome were defined according to status with respect to cardiovascular disease at baseline (yes vs. no), age (<75 vs. ≥75 years), sex, race (black vs. non-black), and baseline systolic blood pressure in three levels (≤ 132 mm Hg, >132 to <145 mm Hg, and ≥145 mm Hg).

### Statistics

For database management and statistical analysis, we used SAS software version 9.4 (SAS Institute Inc, Cary, NC). Significance was a 2-tailed α-level of ≤ 0.05. Means and proportions were compared using the large-sample z-test and the χ2 statistic, respectively. Comparisons of continuous variables among the six eGFR and albuminuria phenotypes were performed by one-way ANOVA.

Kaplan-Meier survival probabilities were estimated according to CKD and albuminuria phenotypes, and differences were analyzed by the log-rank test in four subgroups. Cox proportional hazards analysis was used to compute the hazard ratio (HR) with 95% CI and cumulative survival rate according to CKD phenotypes in the total population as well as in the standard-therapy group analyzed separately, using the UACR <30 mg/g combined with eGFR ≥60 mL/min/1.73 m^2^ as reference group, adjusted for baseline age, sex, race, smoking, history of CVD, BMI, systolic and diastolic blood pressures, and glucose and serum lipid levels.

## Results

This study included 8,886 people with hypertensive patients who were enrolled into the SPRINT clinical trial. The median follow-up duration was 3.3 (25th−75th percentile, 2.8–3.8) years. Characteristics of the study participants at baseline were summarized in Table 1. The prevalence of chronic kidney disease phenotypes was 10.8% for albuminuric non-CKD, 16.8% for non-albuminuric CKD, and 8.6% for albuminuric CKD. Compared with non-albuminuric non-CKD group, individuals with non-albuminuric CKD 3b/4/5 (eGFR <45 mL/min/1.73 m^2^) were older (73.1 vs. 66.1 years), more frequently women (51.4 vs. 33.6%), and nonsmokers (49.1 vs. 43.6%), had lower BMI (29.6 vs. 30.0 Kg/m^2^), and systolic (136.9 vs. 139.0 mmHg) and diastolic blood pressure (72.0 vs. 79.1 mmHg), had decreased eGFR (38.8 vs. 80.1 ml/min/1.73 m^2^), but had higher levels of UACR (11.9 vs. 9.5 mg/dl) and higher 10-year risk (20.3 vs.19.2%).

**Table 1:** Baseline characteristics of study participants by baseline CKD and albuminuria status.

**Variables**	**Overall**	**Non-albuminuria, eGFR (≥60)**	**Non-albuminuria, eGFR (45–59)**	**Non-albuminuria, eGFR (< 45)**	**Albuminuria, eGFR (≥60)**	**Albuminuria, eGFR (45–59)**	**Albuminuria, eGFR (< 45)**	***P^#^***
*n*	8,886	5,671 (63.8)	1,048 (11.8)	444 (5.0)	955 (10.8)	382 (4.3)	386 (4.3)	
Age	67.9 ± 9.4	66.1 ± 8.8	71.9 ± 8.9	73.1 ± 9.5^**^	68.5 ± 9.6	73.2 ± 9.7	71.8 ± 10.7	< 0.001
Female, *n* (%)	3,135 (35.3)	1,906 (33.6)	425 (40.6)	228 (51.4)^**^	314 (32.9)	112 (29.3)	150 (38.9)	< 0.001
**Race**, ***n*** **(%)**								
White	5,102 (57.4)	3,273 (57.7)	610 (58.2)	230 (51.8)	578 (60.5)	220 (57.6)	191 (49.5)	< 0.001
Black	2,682 (30.2)	1,587 (28.0)	364 (34.7)	179 (40.3)	261 (27.3)	128 (33.5)	163 (42.2)	< 0.001
Hispanic	938 (10.6)	696 (12.3)	58 (5.5)	31 (7.0)	99 (10.4)	28 (7.3)	26 (6.7)	< 0.001
Other	164 (1.8)	115 (2.0)	16 (1.5)	4 (0.9)	17 (1.8)	6 (2.1)	6 (1.6)	< 0.001
**Smoking status**, ***n*** **(%)**								
Never smoked	3,904 (43.9)	2,473 (43.6)	506 (48.3)	218 (49.1)	382 (40.0)	165 (43.2)	160 (41.5)	< 0.001
Former smoker	3,785 (42.6)	2,364 (41.7)	450 (42.9)	189 (42.6)	413 (43.3)	186 (48.7)	183 (47.4)	< 0.001
Current smoker	1,187 (13.4)	827 (14.6)	91 (8.7)	37 (8.3)	160 (16.8)	29 (7.6)	43 (11.1)	< 0.001
BMI, Kg/m^2^	29.9 ± 5.8	30 ± 5.7	29.5 ± 5.6	29.6 ± 6^*^	30.2 ± 6.2	29.7 ± 6.2	28.7 ± 5.8	< 0.001
SBP, mmHg	139.7 ± 15.6	139 ± 15	137.9 ± 15.3	136.9 ± 15.8^**^	144.7 ± 16.8	143.3 ± 16.5	141.7 ± 17.3	< 0.001
DBP, mmHg	78.1 ± 12	79.1 ± 11.3	75.1 ± 11.7	72.0 ± 12.4^*^	80.3 ± 12.9	76.7 ± 13.9	75.8 ± 13.2	< 0.001
Glucose, mg/dL	98.9 ± 13.6	99 ± 13.3	98.3 ± 13.2	97.2 ± 12.2	100.5 ± 16.3	98.6 ± 12.4	96.6 ± 14.7	< 0.001
TC, mg/dL	189.9 ± 41.1	191.9 ± 40.7	188.1 ± 40	186.4 ± 39.7	187.8 ± 43.3	180.3 ± 41.2	183.9 ± 41.1	< 0.001
HDL_C, mg/dL	53.3 ± 15.2	52.7 ± 14.1	53.8 ± 14.8	53.3 ± 15	52.5 ± 16.1	51.8 ± 14	52.8 ± 15.9	0.10
TG, mg/dL	126 ± 84	125.4 ± 83.5	120.5 ± 84.8	127.1 ± 65.9	131.1 ± 87.8	124.1 ± 82.2	137.2 ± 97.6	< 0.001
UACR, mg/dl	42.6 ± 166.4	9.5 ± 6	9.8 ± 6.5	11.9 ± 7.1^**^	132.8 ± 290	176.3 ± 265	298.3 ± 487.7	< 0.001
eGFR, ml/min/1.73 m^2^	71.6 ± 20.7	80.1 ± 15.8	53.1 ± 6.2	38.8 ± 6.3^**^	79.6 ± 18.2	52.3 ± 5.8	34.6 ± 7.7	< 0.001
10-year risk (%)	20.1 ± 10.9	19.2 ± 10.0	20.9 ± 11.3	20.3 ± 12.6^*^	22.4 ± 11.4	24.4 ± 12.2	22.5 ± 13.5	< 0.001

Values are mean ± SD, or number of participants (%).

# The *p*-value showed the differences in means or proportions of risk factors across six groups were compared using ANOVA or x^2^ tests as appropriate.

* *P* < 0.05;

** *P* < 0.01 compared to non-albuminuric group with eGFR ≥60 ml/min/1.73 m^2^.

**Figure 2:**
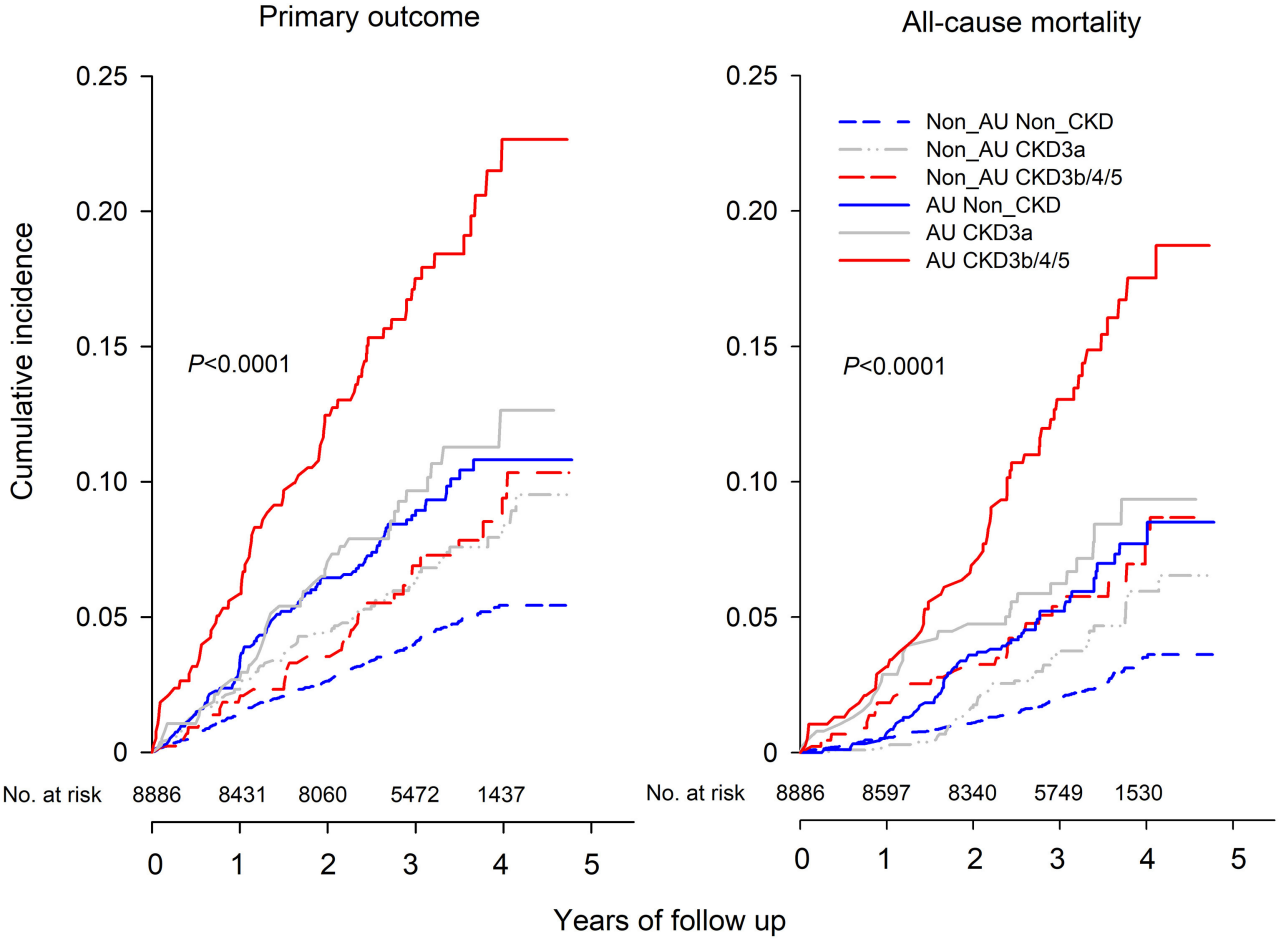
Kaplan-Meier curve of cumulative incidence for primary outcome **(left)** and all-cause mortality **(right)** according to the GFR and Albuminuria categories at baseline. The six subgroups included Non_AU Non_CKD (UACR < 30 mg/g and eGFR ≥60 mL/min/1.73 m^2^), Non_AU CKD3a (UACR < 30 mg/g and eGFR of 45–59 mL/min/1.73 m^2^), Non_AU CKD3b/4/5 (UACR < 30 mg/g and eGFR of < 45 mL/min/1.73 m^2^), AU Non_CKD (UACR ≥30 mg/g and eGFR ≥60 mL/min/1.73 m^2^), AU CKD 3a (UACR ≥30 mg/g and eGFR of 45–59 mL/min/1.73 m^2^), and AU CKD 3b/4/5 (UACR ≥30 mg/g and eGFR of < 45 mL/min/1.73 m^2^).

During a median follow-up of 3.3 years, 547 (6.2%) people developed the primary outcome, and 352 (3.9%) and 99 (1.1%) people died of any cause and of cardiovascular causes, respectively. The cumulative survival rates for these outcomes differed among the six CKD phenotypes, non-albuminuric non-CKD, non-albuminuric CKD 3a, non-albuminuric CKD 3b/4/5, albuminuric non-CKD, albuminuric CKD 3a, and albuminuric CKD 3b/4/5. Compared with non-albuminuric non-CKD, patients with eGFR < 45 ml/min/1.73 m^2^ or UACR≥30 mg/g had significantly lower survival rate of primary outcome and all-cause and cardiovascular mortality, and patients with albuminuric CKD 3b/4/5 had the lowest survival rate of all outcomes (Figure 2).

In the adjusted Cox regression models, we investigated the risks associated with other CKD phenotypes compared with non-albuminuric non-CKD. Patients with albuminuric CKD 3b/4/5 had increased likelihood of the primary outcome, of total mortality, and of CVD mortality in the total population as well as in the intensive- and standard-therapy group analyzed separately (HR from 2.94 to 6.47). Albuminuric non-CKD was associated with an increase in the primary outcome and in all-cause mortality in the total population as well as in the intensive- and standard-therapy group analyzed separately (HR from 1.77 to 2.16).

Non-albuminuric CKD, generally, was neither associated with a significant increase in adverse outcomes in the total population nor in the standard- and intensive-therapy groups analyzed separately (Table 2). Furthermore, no prognostic significance was seen with non-albuminuric CKD on primary outcome across the pre-specified subgroups (Table 3). However, in the non-albuminuric CKD 3b/4/5 group (*n* = 444), there was a suggestion of increased both all-cause and CVD mortality which was statistically significant in the intensive-treatment subset (HR [95% CI], 2.55 [1.43–4.56] and 6.46 [2.57–16.3], respectively), but not in the standard-treatment subset (Table 2). When a sub analysis of the components of the composite CVD end-points was further conducted, only heart failure significantly increased in the non-albuminuric CKD 3b/4/5 group in intensive-treatment subset (HR [95% CI], 5.79 (2.34–14.4) but not in standard-treatment subset (Supplementary Table S1).

**Table 2:** Risk of Primary outcomes and total and cardiovascular mortality by baseline CKD and albuminuria status.

**Outcomes**	**No. of events**	**Non-albuminuria, eGFR (≥60),** ***n*** **= 5,617**	**Non-albuminuria, eGFR (45–59), *n* = 1,048**	**Non-albuminuria, eGFR (< 45), *n* = 444**	**Albuminuria, eGFR (≥60),** ***n*** **= 955**	**Albuminuria, eGFR (45–59),** ***n*** **= 382**	**Albuminuria, eGFR (< 45), *n* = 386**
Total population^*^							
Primary outcome	547	Reference	1.28 (0.98–1.68)	1.22 (0.84–1.79)	**1.82 (1.42**–**2.33)**	**1.69 (1.19**–**2.39)**	**3.38 (2.56**–**4.46)**
All deaths	352	Reference	1.30 (0.92–1.84)	**1.78 (1.16**–**2.72)**	**2.07 (1.51**–**2.83)**	**1.95 (1.29**–**2.96)**	**4.14 (2.98**–**5.73)**
CVD deaths	99	Reference	0.62 (0.26–1.48)	**2.95 (1.50**–**5.80)**	1.49 (0.79–2.81)	1.39 (0.58–3.34)	**5.38 (3.05**–**9.47)**
**Intensive group** ^#^							
Primary outcome	235	Reference	1.36 (0.90–2.06)	**1.75 (1.05**–**2.92)**	**1.77 (1.20**–**2.62)**	**1.86 (1.11**–**3.11)**	**4.07(2.69**–**6.17)**
All deaths	149	Reference	1.40 (0.82–2.38)	**2.55 (1.43**–**4.56)**	**2.16 (1.34**–**3.48)**	1.63 (0.82–3.24)	**4.59 (2.77**–**7.58)**
CVD deaths	36	Reference	-	**6.46 (2.57**–**16.3)**	2.05 (0.77–5.49)	0.70 (0.09–5.48)	**6.47 (2.45**–**17.10)**
**Standard group** ^#^							
Primary outcome	312	Reference	1.23 (0.87–1.75)	0.86 (0.48–1.53)	**1.93 (1.39**–**2.67)**	**1.61 (1.00**–**2.61)**	**2.94 (2.02**–**4.27)**
All deaths	203	Reference	1.24 (0.79–1.95)	1.25 (0.66–2.38)	**2.10 (1.39**–**3.18)**	**2.17 (1.28**–**3.69)**	**3.93 (2.56**–**6.04)**
CVD deaths	63	Reference	0.93 (0.38–2.28)	1.18 (0.35–4.00)	1.29 (0.56–2.97)	**1.72 (0.64**–**4.62)**	**5.17 (2.57**–**10.40)**

* Adjusted for therapy group, age, sex, race, smoking, history of CVD, BMI, systolic and diastolic blood pressures, and glucose and serum lipid levels.

# Adjusted for age, sex, race, smoking, history of CVD, BMI, systolic and diastolic blood pressures, and glucose and serum lipid levels.

Albuminuria was defined as a urinary albumin-to-creatinine (ACR) ratio ≥30 mg/g.

CKD was defined as eGFR < 60 mL/min/1.73 m^2^. The primary outcome is a composite of fatal CVD, MI, stroke, heart failure, and non-MI acute coronary syndrome.

The bold values means the significance of difference with the reference.

**Table 3:** Risk of primary outcomes by baseline CKD and albuminuria status according to subgroups.

	**Non-albuminuria, eGFR (≥60)**	**Non-albuminuria, eGFR (45–59)**	**Non-albuminuria, eGFR(< 45)**	**Albuminuria**
**Previous CVD**				
No (*n* = 7,098)	Reference	1.21 (0.89–1.68)	1.59 (1.03–2.41)	2.18 (1.70–2.78)
Yes (*n* = 1,802)	Reference	0.96 (0.46–1.58)	0.82 (0.42–1.58)	1.85 (1.32–2.55)
*p*-value for interaction^#^	-	0.70	0.52	0.35
**Age**				
< 75 y (*n* = 6,359)	Reference	1.08 (0.72–1.61)	1.59 (0.92–2.67)	**2.24 (1.68**–**2.89)**
≥75 y (*n* = 2,541)	Reference	1.09 (0.75–1.59)	0.98 (0.58–1.61)	**1.88 (1.28**–**2.48)**
*p*-value for interaction^#^		0.72	0.77	0.88
**Sex**				
Female (*n* = 3,141)	Reference	1.43 (0.92–2.25)	1.28 (0.70–2.38)	**2.44 (1.66**–**3.56)**
Male (*n* = 5,759)	Reference	0.93 (0.62–1.32)	1.21 (0.75–1.88)	**1.92 (1.50**–**2.42)**
*p*-value for interaction^#^		0.14	0.88	0.22
**Race**				
Black (*n* = 2,828)	Reference	1.29 (0.68–2.28)	1.32 (0.50–3.18)	2.92 (2.01–4.12)
Non-black (*n* = 6,072)	Reference	1.04 (0.78–1.38)	1.09 (0.76–1.66)	1.78 (1.42–2.20)
*p*-value for interaction^#^		0.78	0.80	0.06
**Systolic blood pressure**				
≤ 132 mm Hg (*n* = 2,978)	Reference	1.12 (0.72–1.75)	1.17 (0.60–2.28)	2.33 (1.60–3.38)
>132 to < 145 mm Hg (*n* = 3,083)	Reference	1.09 (0.70–1.69)	1.10 (0.60–2.04)	1.89 (1.35–2.67)
≥145 mm Hg (*n* = 2,839)	Reference	1.15 (0.72–1.84)	1.51 (0.85–2.69)	2.09 (1.52–2.88)
*p*-value for interaction^#^		0.67	0.43	0.66

# Adjusted for age, sex, race, smoking, history of CVD, BMI, systolic and diastolic blood pressures, and glucose and serum lipid levels. Albuminuria was defined as a urinary albumin-to-creatinine (UACR) ratio ≥30 mg/g.

The bold values means the significance of difference with the reference.

Comparisons of primary outcome and mortality in the subgroups defined by the presence or absence of albuminuria and by eGFR ≥60, ≥45 to < 60, or < 45 showed reduction in both outcomes in participants randomized intensive vs. standard BP goals in trial participants with normoalbuminuria and eGFR ≥60, with normoalbuminuria and eGFR ≥45 to < 60, and with albuminuria in all the eGFR subgroups (Supplementary Table S1). However, among trial participants with eGFR < 45 and normoalbuminuria the likelihood of mortality was 1.39-fold (95% CI 0.65–2.99) greater with the intensive BP goal than that with the standard BP goal (Supplementary Table S2).

## Discussion

This *post-hoc* analysis of the SPRINT trial found that non-albuminuric CKD did not have higher risk of primary outcome and mortality in hypertensive patients alone. However, increased mortality was shown in the intensive treatment subset if the eGFR < 45 mL/min/1.73 m^2^. Generally, increased albuminuria, rather than decreased eGFR, might be the main driver for adverse outcomes in hypertensive patients.

Our study exhibited similar results to those conducted in persons with diabetes mellitus, and found that non-albuminuric CKD did not have higher risk of primary outcome and mortality. One study of 2,953 Japanese patients with type 2 diabetes and estimated glomerular filtration rate (eGFR) ≥30 mL/min/1.73 m^2^ found that the risks of death or CVD were not higher in those with non-albuminuric CKD than those with non-CKD (adjusted hazard ratio, 1.02 [95% CI 0.66–1.60]) (11). Another study conducted in 1,908 participants with diabetes and reduced eGFR found that the absence of albuminuria carried a much lower risk for ESKD, CKD progression, or rapid decline in eGFR compared to that in patients with albuminuria (20). However, among persons with type 2 diabetes enrolled in the Action to Control Cardiovascular Risk in Diabetes (ACCORD) study (10), patients with non-albuminuric CKD had higher risk of death or major cardiovascular events than those in non-CKD patients. Nevertheless, the risk of those with non-albuminuric CKD was much lower than that of those with albumuniuric CKD and albuminuric non-CKD. Our study of non-diabetic hypertensive patients found similar results:persons with nonalbuminuric reduced eGFR did not have higher risk of adverse outcomes than those in hypertensive patients with normal glomerular filtration. However, our study firstly found that non-albuminuric CKD 3b/4/5 did have higher risk of all-cause and cardiovascular mortality. The risk was only seen in heart failure when the composite CVD components was further analyzed, similar to the original SPRINT study (19). However, intensive BP-lowering was associated with greater rather than lower risk of adverse outcome.

There are several explanations for these results. Firstly, non-albuminuria with reduced eGFR could happen in histopathologically diagnosed tubulointerstitial disease which is known by no significant proteinuria, no clinically evident edema, and no known cause of chronic renal failure (21). Secondly, it is possible that non-albuminuric reduced GFR mainly reflects an age-related decline in renal function, rather than a primary renal disease. In this study, non-albuminuric CKD showed about 8 years older than the reference group, though slightly younger than albuminuric CKD.

Population-based studies consistently showed that prevalence of CKD depends strongly on age, with prevalence increased approximately 10-fold between young adulthood and middle age in the US Kidney Early Evaluation Program (22). Thirdly, another possibility is that the non-albuminuric reduced GFR group is enriched with people with who respond particularly favorably to RAAS blockade, leading to normalization of urinary albumin-to-creatinine ratio and increased serum creatinine (23).

Although risk for end-stage kidney disease was very low in people with non-albuminuric CKD, reduced eGFR remains an important marker for all-cause mortality and CVD (24). Studies have showed that reduced eGFR is an important prognostic factor in total mortality and cardiovascular events, inspective of albuminuria, in general population or uncomplicated hypertensive patients (24) or resistant hypertensive patients (25).

Our study should be interpreted within the context of its strengths and limitations. The strengths of our study include a large sample size and long follow up, which enable us to classify the study population into six subgroups for detailed analysis. Some limitations of this analysis should be discussed. Our study was a *post-hoc* analysis of a highly selected group of individuals with prior CVD, and the results should be validated in unselected hypertensive populations. The study population was classified into six subgroups, which might be underpowered to conduct subgroup analysis, such as greater HRs and wide range of CIs for NACKD predicting CVD mortality. Our study did not take frequent serum creatinine sampling, and we could not compare the rate of CKD progression between non-albuminuric CKD and other groups. The study did not use the KDIGO definition of CKD, as only one measure of serum creatinine and albumninuria at baseline was conducted.

## Conclusion

Non-albuminuric CKD did have higher risk of all-cause and CVD mortality only if the eGFR <45 mL/min/1.73 m^2^. Increased albuminuria conferred higher risk of primary outcome and all-cause mortality irrespective the levels of eGFR.

## Data availability statement

The datasets presented in this study can be found in online repositories. The names of the repository/repositories and accession number(s) can be found in the article/Supplementary material.

## Ethics statement

Written informed consent was obtained from the individual(s) for the publication of any potentially identifiable images or data included in this article.

## Author contributions

C-SS, JT, and ZB had full access to all the data in the study, take responsibility for the accuracy of the data analysis, and participated in the study design and paper revision. DW, JY, and YC performed the studies and drafted the manuscript. SS and YY helped with the statistical analysis. YM and WW checked the accuracy of the analysis and involved in review the English language and grammar. All the authors read and approved the final manuscript.
