# Supplementary material for: CVD risk in non-albuminuric chronic kidney disease in hypertensive, non-diabetic subjects: A post-hoc analysis from SPRINT
Source: Front Cardiovasc Med. 2022 Dec 7;9:977938. doi: 10.3389/fcvm.2022.977938 (PMC9768444; doi:10.3389/fcvm.2022.977938)
Supplement: Supplementary file 1 [file Table_1.DOCX]

*Frontiers in Cardiovascular Medicine*

**Supplemental Material**

This appendix formed part of the original submission and has been peer reviewed.
Supplement to: CVD Risk in Nonalbuminuric Chronic Kidney Disease in Hypertensive, Non-diabetic Subjects: A Post Hoc Analysis from SPRINT

**Tables 2**

**Table S1 . Risk of components of the composite CVD end-points by baseline CKD and albuminuria status**

| Outcomes | No. of events | Nonalbuminuria,  eGFR (≥60), n=5617 | Nonalbuminuria,  eGFR (45-59), n=1048 | Nonalbuminuria,  eGFR (<45),  n=444 | Albuminuria,  eGFR (≥60), n=955 | Albuminuria,  eGFR (45-59), n=382 | Albuminuria,  eGFR (<45), n=386 |
| --- | --- | --- | --- | --- | --- | --- | --- |
| Total population* |  |  |  |  |  |  |  |
| Myocardial infarction | 207 | Reference | 1.25 (0.82-1.92) | 0.67 (0.31-1.45) | **1.81 (1.23-2.68)** | 1.47 (0.83-2.61) | **2.44 (21.51-3.94)** |
| Acute coronary syndrome | 77 | Reference | 0.86 (0.42-1.80) | 0.88 (0.31-2.51) | 1.34 (0.70-2.56) | 0.23 (0.03-1.71) | 1.36 (0.53-3.49) |
| Stroke | 129 | Reference | 0.95 (0.52-1.72) | 1.36 (0.66-2.79) | 1.64 (0.98-2.74) | 1.51 (0.74-3.09) | **3.11 (1.78-5.45)** |
| Heart failure | 159 | Reference | **2.04 (1.23-3.39)** | **2.80 (1.55-5.07)** | **2.82 (1.72-4.62)** | **3.34 (1.86-6.01)** | **7.97 (4.98-12.75)** |
| Intensive group# |  |  |  |  |  |  |  |
| Myocardial infarction | 94 | Reference | 1.50 (0.79-2.86) | 0.96 (0.33-2.74) | **2.33 (1.32-4.12)** | 2.06 (0.94-4.52) | **3.57 (1.82-7.01)** |
| Acute coronary syndrome | 39 | Reference | 1.27 (0.50-3.23) | 0.52 (0.07-3.96) | 1.22 (0.49-3.06) | **0.47 (0.06-3.52)** | **1.63 (0.47-5.65)** |
| Stroke | 59 | Reference | 1.07 (0.48-2.37) | 1.58 (0.63-3.93) | 0.94 (0.39-2.29) | 0.90 (0.27-3.02) | 2.34 (0.95-5.78) |
| Heart failure | 60 | Reference | 1.50 (0.52-4.32) | **5.79 (2.34-14.4)** | **3.44 (1.39-8.51)** | **6.54 (2.68-16.0)** | **17.7 (8.22-38.3)** |
| Standard group# |  |  |  |  |  |  |  |
| Myocardial infarction | 113 | Reference | 1.10 (0.62-1.94) | 0.48 (0.15-1.54) | **1.49 (0.86-2.57)** | **1.09 (0.46-2.58)** | **1.74 (0.87-3.49)** |
| Acute coronary syndrome | 38 | Reference | 0.48 (0.14-1.62) | 1.01 (0.29-3.50) | 1.58 (0.64-3.90) | **-** | **1.22 (0.28-5.27)** |
| Stroke | 70 | Reference | 0.85 (0.35-2.07) | 1.06 (0.32-3.55) | **2.52 (1.32-4.79)** | **2.30 (0.92-5.70)** | **4.03 (1.95-8.34)** |
| Heart failure | 99 | Reference | 2.22 (1.24-3.97) | 1.73 (0.75-4.00) | **2.71 (1.49-4.90）** | 2.06 (0.89-4.78) | **4.91 (2.62-9.20)** |

*Adjusted for therapy group, age, sex, race, smoking, history of CVD, BMI, systolic and diastolic blood pressures, and glucose and serum lipid levels.

#Adjusted for age, sex, race, smoking, history of CVD, BMI, systolic and diastolic blood pressures, and glucose and serum lipid levels.

Albuminuria was defined as a urinary albumin-to-creatinine (ACR) ratio≥30 mg/g.

CKD was defined as eGFR <60 mL/min/1.73 m^2^. The primary outcome is a composite of fatal CVD, MI, stroke, heart failure, and non-MI acute coronary syndrome.

**Table S2. Risk of Primary Outcomes and Total and Cardiovascular mortality by baseline CKD and albuminuria status**

|  | eGFR, ≥60 | | | eGFR, 45-59 | | | | eGFR, <45 | | | |
| --- | --- | --- | --- | --- | --- | --- | --- | --- | --- | --- | --- |
|  | Intensive | Standard | Intensive/standard ratio | Intensive | | Standard | Intensive/standard ratio | Intensive | Standard | Intensive/standard ratio |  |
| Primary outcome |  |  |  | |  |  |  |  |  |  |  |
| Nonalbuminuria | 3.48 | 5.35 | 0.65 (0.50-0.85) | 5.93 | | 6.58 | 0.90 (0.57-1.42) | **8.40** | **7.23** | **1.16 (0.59-2.27)^a^** |  |
| Albuminuria | 7.51 | 10.0 | 0.75 (0.47-1.19) | 7.73 | | 12.5 | 0.62 (0.32-1.18) | 17 | 18.7 | 0.91 (0.55-1.51) |  |
| All-cause deaths |  |  |  |  | |  |  |  |  |  |  |
| Nonalbuminuria | 2.03 | 2.86 | 0.71 (0.51-0.99) | 2.96 | | 4.82 | 0.61 (0.34-1.10) | **7.14** | **5.11** | **1.39 (0.65-2.99)^b^** |  |
| Albuminuria | 5.36 | 7.27 | 0.74 (0.43-1.26) | 4.83 | | 7.87 | 0.61 (0.27-1.37) | 12 | 17.17 | 0.70 (0.40-1.22) |  |

**^a^*P*=0.12 vs. the intensive/standard ratio in nonalbuminuria with eGFR≥60. ^b^P=0.06 vs. the intensive/standard ratio in nonalbuminuria with eGFR≥60.**

**Adjusted rate (%) of events by eGFR and albuminuria status and ratio (with 95% CI) of outcomes with intensive vs. standard treatment.**

Adjusted for age, sex, race, smoking, history of CVD, BMI, systolic and diastolic blood pressures, and glucose and serum lipid levels.

Albuminuria and Nonalbuminuria defined as urinary albumin-to-creatinine (ACR) ratio≥30 and <30 mg/g, respectively. The primary outcome is a composite of fatal CVD, MI, stroke, heart failure, and non-MI acute coronary syndrome.
